# Combined amino acid PET-MRI for identifying recurrence in post-treatment gliomas: together we grow

**DOI:** 10.1186/s41824-021-00109-y

**Published:** 2021-08-18

**Authors:** Shumyla Jabeen, Arpana Arbind, Dinesh Kumar, Pardeep Kumar Singh, Jitender Saini, Nishanth Sadashiva, Uday Krishna, Arivazhagan Arimappamagan, Vani Santosh, Chandana Nagaraj

**Affiliations:** 1 Sher-i-Kashmir Institute of Medical Sciences, Srinagar, Kashmir 190001 India; 2 Department of Neuroimaging and Interventional Radiology, National Institute of Mental Health and Neurosciences, Bengaluru, Karnataka 560029 India; 3 Department of Neurosurgery, National Institute of Mental Health and Neurosciences, Bengaluru, Karnataka 560029 India; 4 Department of Radiation Oncology, Kidwai Memorial Institute of Oncology, Bengaluru, Karnataka 560029 India; 5 Department of Neuropathology, National Institute of Mental Health and Neurosciences, Bengaluru, Karnataka 560029 India

**Keywords:** Amino acid PET, Gliomas, Radiation necrosis, Recurrence, Perfusion, Diffusion

## Abstract

**Abstract:**

**Purpose:**

The aim of this study is to compare the diagnostic accuracy of amino acid PET, MR perfusion and diffusion as stand-alone modalities and in combination in identifying recurrence in post-treatment gliomas and to qualitatively assess spatial concordance between the three modalities using simultaneous PET-MR acquisition.

**Methods:**

A retrospective review of 48 cases of post-treatment gliomas who underwent simultaneous PET-MRI using C11 methionine as radiotracer was performed. MR perfusion and diffusion sequences were acquired during the PET study. The following parameters were obtained: TBR_max_, TBR_mean_, SUV_max_, and SUV_mean_ from the PET images; rCBV from perfusion; and ADC_mean_ and ADC_ratio_ from the diffusion images. The final diagnosis was based on clinical/imaging follow-up and histopathology when available. ROC curve analysis in combination with logistic regression analysis was used to compare the diagnostic performance. Spatial concordance between modalities was graded as 0, 1, and 2 representing discordance, < 50% and > 50% concordance respectively.

**Results:**

There were 35 cases of recurrence and 13 cases of post-treatment changes without recurrence. The highest area under curve (AUC) was obtained for TBR_max_ followed by rCBV and ADC_ratio_. The AUC increased significantly with a combination of rCBV and TBR_max_. Amino acid PET showed the highest diagnostic accuracy and maximum agreement with the final diagnosis. There was discordance between ADC and PET in 22.9%, between rCBV and PET in 16.7% and between PET and contrast enhancement in 14.6% cases.

**Conclusion:**

Amino acid PET had the highest diagnostic accuracy in identifying recurrence in post-treatment gliomas. Combination of PET with MRI further increased the AUC thus improving the diagnostic performance.

## Introduction

Gliomas are the most common primary brain neoplasms (Ostrom et al., 2018). Maximal safe surgical excision with adjuvant chemoradiotherapy is the mainstay of treatment for high grade gliomas (grade III and IV). However, recurrence rates continue to remain high with poor overall survival despite treatment especially in case of glioblastoma (Weathers & Gilbert, 2015; Stupp et al., 2005a). This mandates stringent post-treatment clinical and imaging surveillance. Magnetic resonance imaging (MRI) is indispensable for assessment of disease burden and response following therapy. Recent literature has brought focus on the various treatment effects in brain due to radiation therapy, chemotherapy, and immunotherapy, the imaging features of which can often resemble tumor recurrence. Distinguishing recurrence from treatment-related changes as well as identifying recurrence in a background of such changes is extremely challenging and has profound prognostic and therapeutic implications. There is also a need to set out robust criteria for enrollment in clinical trials and evaluation of efficacy of new emerging therapies. Over several years, the consensus Response Assessment in Neuro-Oncology (RANO) criteria based on conventional MRI and clinical assessment have been used toward achieving this end (Wen et al., 2017). Although, there are guidelines for ruling out pseudo-progression with these criteria, this requires a waiting period of 3 months following chemoradiotherapy during which a definitive diagnosis cannot be made (Wen et al., 2017). Besides, recurrent tumor can often co-exist with radiation necrosis (Sugahara et al., 2000) and the two cannot be resolved on conventional imaging alone. Advanced MRI techniques like perfusion, spectroscopy, and quantification of diffusion parameters have shown to be useful in detecting progressive disease as highlighted in several earlier studies. However, at the cost of various challenges in interpretation due to overlapping parameters (Seeger et al., 2013; Keunen et al., 2014; Heiss et al., 2011). Molecular imaging, which reflects the tumoral physiologic milieu, acts as a problem-solving tool complementary to MRI. FDG-PET has an established role in post-treatment imaging of gliomas (Wang et al., 2015; Nozawa et al., 2015; Nihashi et al., 2013). However, increased uptake by inflammatory cells and high background uptake by the normal brain parenchyma can lead to a false diagnosis (Nihashi et al., 2013). In this regard, PET imaging with alternate metabolites like C-11 methionine may be advantageous in view of reduced normal parenchymal uptake. Also, discordance between MR perfusion and FDG-PET was demonstrated in an earlier study highlighting the different functional parameters which they reflect (Jena et al., 2017). Besides, in most of the early studies based on advanced MRI and PET imaging, the two studies were performed at different time points thus adding to the complexity of the results. In this study, we compared the diagnostic accuracies of amino acid PET, MR perfusion, and diffusion as standalone modalities and in combination in identifying recurrence in treated gliomas using simultaneous PET-MR acquisition. In addition, a qualitative assessment of spatial concordance between increased metabolic uptake on PET, elevated perfusion on dynamic susceptibility contrast (DSC) MRI, and restricted diffusion on diffusion-weighted imaging was performed.

## Material and methods

### Type of study

This single institute retrospective study was carried out at a dedicated quaternary care center providing neurosurgical and neuro-imaging services for patients with brain tumors. Written informed consent was obtained from all subjects prior to imaging. The study was approved by institutional ethics committee review board.

### Subjects

All cases of histopathologically proven glial tumors who had undergone surgical resection followed by fractionated radiotherapy with or without standard chemotherapy with temozolomide who underwent simultaneous amino acid PET-MRI with suspected recurrence between January 2019 and March 2020 were included in the study. Exclusion criteria included non-glial primary brain tumors, metastatic lesions, and standard contraindications for PET and/or MRI like pregnancy, end-stage renal disease, and presence of a cardiac pacemaker or MRI incompatible metallic implants. Molecular biomarker data were obtained from histopathology reports of the primary tumor at initial biopsy or resection. Radiation therapy to a total dose of 60 Gy in 30 fractions was usually initiated within 6 weeks after surgery. Temozolomide was given concurrently and sequentially, per Stupp et al. (Stupp et al., 2005b).

### Imaging technique

All patients underwent simultaneous amino-acid PET-MR imaging on a 3 Tesla SIEMENS, Biograph mMR scanner (Erlangen, Germany). All patients were fasted 4–6 h prior to scanning for baseline stable metabolic conditions. On the day of imaging, all patients were injected 360–378 MBq of C11 methionine on the table through IV cannula. Simultaneous acquisition of PET images was performed along with UTE MR attenuation correction sequence (MRAC) along with other standard and advanced MRI sequences for 40 min in LIST MODE. PET images were acquired using the following parameters: 500 mm FOV, 400 mm anterior-posterior FOV, 1.0 zoom, 3 interactions, 21 subsets, HD PET reconstruction method, and 2.0 mm Gaussian filter.

The following MR sequences were obtained during the PET acquisition: 3D fluid-attenuated inversion recovery (FLAIR)- TR/TE = 5000/385 ms, TI = 1800 ms, voxel size = 1 × 1 × 1 mm, FOV = 256 × 256; axial T1 spin echo-TR/TE = 550/15 ms, slice thickness-4 mm, FOV = 230 × 230; axial T2 spin echo-TR/TE = 5500/92 ms, slice thickness-4 mm, FOV = 230 × 230; axial susceptibility-weighted imaging (SWI)-TR/TE = 27/20 ms, flip angle = 15°, slice thickness = 2 mm, FOV = 230 × 230; axial diffusion-weighted imaging (DWI)-TR/TE = 3900/81 ms, slice thickness-4 mm, FOV = 230 × 230 at *b* values of 50 and 1000.

DSC perfusion was performed after the administration of gadolinium-based contrast agent in a dose of 0.1–0.15 mmol/kg body weight at a rate of 5–6 ml/s using a dual chamber injector connected to a 16-gauge cannula placed in the antecubital vein followed by 25 ml saline chase at the same rate. Echo planar sequence was acquired with parameters as follows TR/TE = 1900/30 ms, flip angle = 90°, slice thickness = 4 mm, FOV = 230 × 230, no. of slices = 25. This was followed by acquisition of post-contrast T1 magnetization prepared rapid gradient-echo (MPRAGE) sequence with TR/TE = 2200/2.33 ms, TI = 900 ms, flip angle = 8°, FOV = 256 × 256, voxel size = 1 × 1 × 1 mm.

### Image analysis

The PET and MRI scans were analyzed by a nuclear medicine specialist and neuroradiologist respectively.

### PET analysis

#### Quantitative ROI analysis

**Fig. 1:**
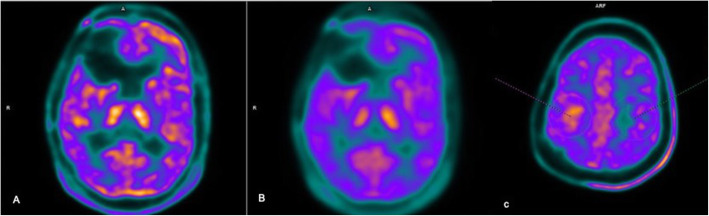
LIST mode UTE MRAC sequence reconstructed PET images (**A**) and images reprocessed on E7 tools SIEMENS for correction of partial volume effects (PVE) (**B**). 3D ROI was drawn semi-automatically using an individually adapted isocontour of the tumor maximum using a standard ROI with a fixed diameter of 1.6 cm centered on the tumor maximum yielding a volume of 2 ml (**C**)

C11 methionine PET images were loaded into SIEMENS SYNGO Via (VB30) workstation after correcting for partial volume effects (PVE) using Siemens E7 tools (Fig. 1A, B). The 3D ROI was drawn semi-automatically using an individually adapted isocontour of the tumor maximum using a standard ROI with a fixed diameter of 1.6 cm centered on the tumor maximum yielding a volume of 2 ml (Fig. 1C). Similar mirror ROI was placed in the contralateral brain parenchyma to calculate the background /normal brain parenchymal uptake (Fig. 1C). The values SUV_max_ and SUV_mean_ were obtained for both tumor and normal brain parenchyma and tabulated. Ratio TBR _max_ and TBR _mean_ (tumor to normal brain/background) were calculated for statistical analysis.

### MRI analysis

#### Quantitative ROI analysis

DSC perfusion, diffusion trace images with ADC maps, and post-contrast images were loaded into Philips Intellispace Portal version 6.0. Perfusion images were processed using the leakage correction algorithm. The colored CBV maps were co-registered with the post-contrast image. After visual assessment, three to four ROIs were drawn in the areas showing elevated perfusion and the ROI with the maximum value was used for further analysis. A mirror ROI was placed in the contralateral normal white matter and the relative CBV ratio obtained. The ADC maps were also co-registered with the post-contrast images and ROI drawn in the same region to obtain the mean ADC value. Another ROI was drawn in the contralateral normal white matter and the ADC ratio calculated.

#### Qualitative grading of diffusion restriction

A qualitative ordinal scale was used to grade the degree of diffusion restriction on ADC maps with grade 1 assigned when the region of interest appeared brighter than the normal white matter, grade 2 when the signal intensity is same as white matter, grade 3 less than white matter, and grade 4 avid, unequivocal diffusion restriction.

#### Qualitative visual assessment for detecting recurrence

A visual analysis of the rCBV maps, ADC maps, and post-contrast images was independently performed to assess for the presence of recurrence without quantification.

#### Spatial concordance between PET, perfusion, and diffusion

The colored rCBV map, ADC map, and post-contrast image each were independently compared to the PET image and the spatial concordance between the area of uptake, elevated rCBV on perfusion, restricted diffusion on ADC map, and enhancement on post-contrast image graded as follows: grade 0-discordance, 1-fair (less than 50%), and 2-moderate (more than 50%) concordance between area of PET uptake and elevated perfusion on rCBV map/restriction on ADC map/enhancement on post-contrast T1 MPRAGE.

#### Final diagnosis

The final diagnosis of recurrence was based on histopathology when available and on clinical and/or imaging follow up for cases where it was not. Disease progression clinically or on imaging was classified as recurrence.

### Statistical analysis

Quantitative variables were expressed as median or mean with standard deviation. Qualitative variables were expressed as percentages. The PET, perfusion, and diffusion parameters between the two groups were compared using the Mann-Whitney *U* test. Receiver operating characteristic (ROC) curve analysis was used to assess the diagnostic performance of each parameter in detecting recurrence. ROC curve analysis in combination with logistic regression analysis was used to measure the area under curve of various combination of parameters. The degree of agreement between perfusion, diffusion, contrast enhancement, PET, and final diagnosis was estimated using the Cohen kappa statistic with values of .01–.20, .21–.40, .41–.60, .61–.80, and .81–1.00 indicating slight, fair, moderate, substantial, and perfect agreement. Spatial concordance between rCBV maps, ADC maps, post-contrast MRI, and PET was expressed as percentages. All analysis was performed on IBM SPSS version 26. A *p* value of less than 0.05 was regarded as significant.

## Results

### Demographics, primary lesion characteristics, and final diagnosis

There were 48 cases of post-treatment glial brain tumors who underwent simultaneous PET-MR imaging during the study period. The mean age at diagnosis was 39.9 ± 12.5 years with range of 8–71 years and median 39.5 years. There were 31 males and 17 females with a male:female ratio of approximately 1.8:1. Patient demographics, tumor characteristics, treatment details, duration following treatment when recurrence was suspected, and follow-up duration after imaging are detailed in Table 1. Post C11 methionine imaging the final diagnosis was considered as recurrence in 35 cases out of which 9 were confirmed on histopathology and 26 had clinical/imaging evidence of progressive disease. Thirteen cases had no evidence of recurrence on clinical/imaging follow-up. The median duration of follow-up after imaging was 7 months ranging between 1 and 14 months (mean 7.5 ± 4 months).

**Table 1:** Median, mean, standard deviation (SD) for PET, perfusion, and diffusion parameters in either group with *p* values obtained using the Mann-Whitney test

Parameters	Recurrence = 35 Mean, Median (SD)	Radiation necrosis = 13 Mean, Median (SD)	***p*** value
**TBR**_**max**_	1.83, 1.59 (1.05)	1.03, 1.02 (0.18)	.000
**TBR**_**mean**_	1.67, 1.57 (1.01)	0.90, 0.95 (0.30)	.000
**SUV**_**max**_	4.72, 4.36 (2.15)	2.66, 2.64 (0.80)	.000
**SUV**_**mean**_	2.44, 2.15 (1.3)	1.52, 1.57 (0.70)	.019
**rCBV**_**ratio**_	2.94, 2.00 (1.8)	0.99, 0.67 (0.94)	.001
**ADC**_**mean**_	0.78, 0.77 (0.14)	0.99, 0.95 (0.38)	.032
**ADC**_**ratio**_	0.99, 0.96 (0.25)	1.49, 1.3 (0.73)	.004

*TBR* tumor to background ratio, *SUV* standard uptake value, *rCBV* relative cerebral blood volume, *ADC* apparent diffusion co-efficient, *SD* standard deviation

### Comparison of quantitative PET, perfusion, and diffusion parameters between recurrence and radiation necrosis

The mean value of each parameter is shown in Table 2. Intergroup comparison using the Mann-Whitney *U* test showed a significant difference between the two groups for all PET, perfusion, and diffusion parameters. SUV_max_, TBR_max_, and TBR_mean_ reached the highest level of significance (*p* value < 0.001) followed by rCBV (*p* value = .001).

**Table 2:** Area under curve values with sensitivity, specificity, positive predictive values (PPV), negative predictive values (NPV), and diagnostic accuracy for important PET, perfusion, and diffusion parameters

Parameter	AUC	CUT-OFF	Sensitivity (%)	Specificity (%)	PPV (%)	NPV (%)	Diagnostic accuracy (%)
**TBR**_**max**_	0.865	1.23	81.8	92.3	91.4	83.5	87.05
**rCBV**_**ratio**_	0.823	1.38	84.8	76.9	78.6	83.5	80.85
**ADC**_**ratio**_	0.776	1.11	78.1	69.2	71.7	75.9	73.65
**ADC**_**grade**_	0.722	> 1	84	46.2	60.9	74.3	65.1

*TBR* tumor to background ratio, *SUV* standard uptake value, *rCBV* relative cerebral blood volume, *ADC* apparent diffusion co-efficient, *AUC* area under the curve

### Diagnostic performance of PET and MRI parameters

**Fig. 2:**
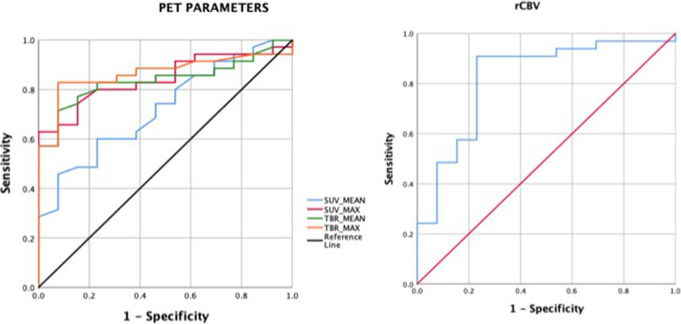
ROC curve analysis for PET parameters on the left and relative CBV on the right. The highest area under curve was obtained for TBR_max_ among the PET parameters followed by SUV_max_, TBR_mean_, and SUV_mean_ in that order

**Fig. 3:**
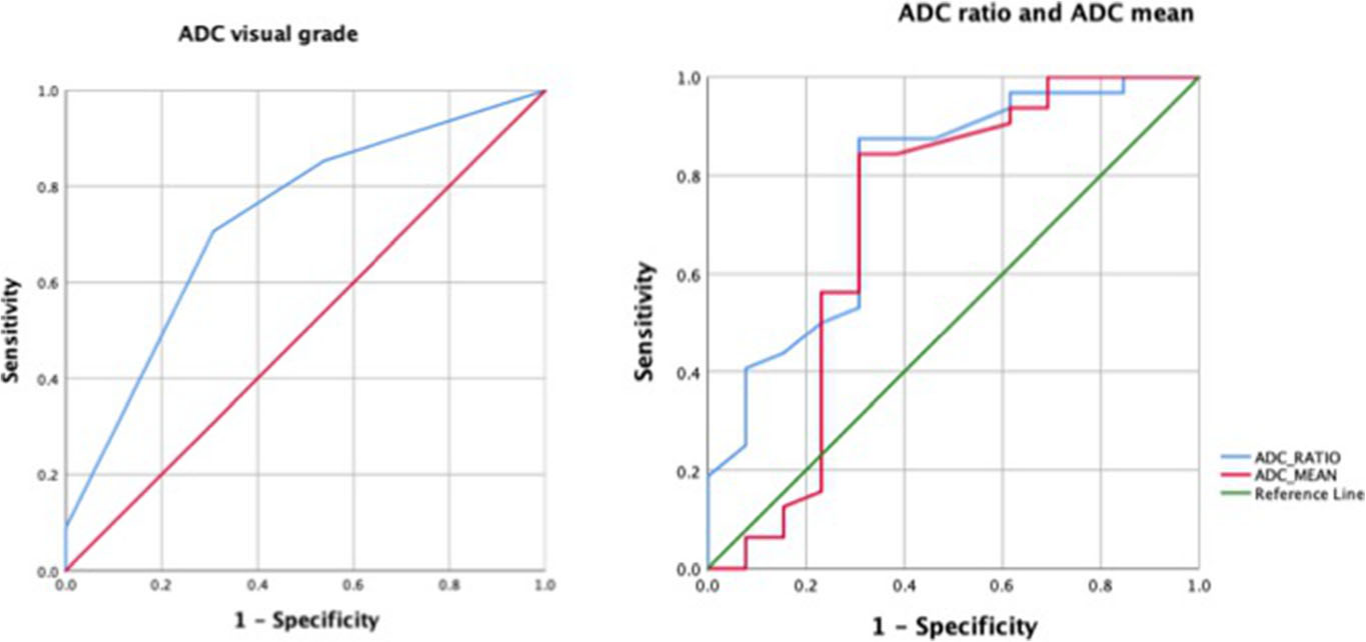
ROC curves for ADC visual grade on the left and ADC_ratio_ as well as mean ADC on the right. The lowest area under curve among the diffusion parameters was that for mean ADC

The ROC curves for each parameter are shown in Figs. 2 and 3 with values detailed in Table 3. Among the PET parameters, the highest area under curve (AUC) was obtained for the TBR_max_ followed by SUV_max_, TBR_mean_, and SUV_mean_ in that order. For MRI, rCBV ratio showed the highest area under curve followed by ADC ratio, qualitative diffusion restriction grade, and mean ADC.

**Table 3:** Patient demographics details, surgical procedure performed, treatment details, and scan duration post follow up

No	Age	Location	Histology	Grade	Molecular subtype	Surgical Details immediate post op imaging not done to assess extent of resection	Chemo/Radiotherapy	Interval between Post treatment completion and PET/MRI	Duration of follow up Post PET/MRI
1	52/M	Right parietal	GBM	IV	IDH wild-type	2009: Partial resection 2009 2017: Gross Total excision of recurrence	2009: EBRT-56 Gy in 28 # with concurrent TMZ 2017: IMRT 60 Gy in 30 #, with concurrent and adjuvant TMZ completed in 2018)	12	12
2	44/M	Right posterior frontal	AA	III	IDH mutant, loss of p53	2012:Gross total excision 2017: Re-exploration and decompression 2017	2012: 56 Gy in 28 # with concurrent TMZ 2017: TMZ completed in 2018	12	13
3	42/M	Right insula	AA	III	IDH mutant	2017: Subtotal resection	2017: EBRT 55 Gy in 31 cycles Concurrent and Adjuvant TMZ till 2018	12	13
4	71/M	Left frontal	GBM	IV	IDH wildtype	2019: Gross total excision	Defaulted treatment		1
5	40/F	Right Parietal	AA	III	IDH mutant	2013: Partial resection	2013: EBRT 60 Gy in 30 # with concurrent TMZ	72	3
6	32/F	Left frontal	AA	III	IDH mutant	2009: Partial resection 2013: Subtotal excision 2018: Decompression with Gross total excision	2014: EBRT 50 Gy 28 # with concurrent TMZ 2018: EBRT 30Gy in 15 cycles with concurrent TMZ	12	1
7	44/M	Right frontal	Astrocytoma	II	IDH mutant	Gross total resection 2018	Defaulted treatment	12	12
8	39/M	Right frontal	AODG	III	FISH not done	2012: Subtotal excision	2012: EBRT 60 Gy 30 # with concurrent TMZ	84	11
9	45/M	Right temporoparietal	GBM	IV	IDH wildtype	2018: Gross Total excision	2018: IMRT with concurrent and adjuvant TMZ (last 2018)	12	4
10	22/M	Right parietooccipital	GBM	IV	IDH wildtype	2013: Gross Total excision	2013: EBRT 46 Gy 25 #, 10 Gy 15 # with concurrent TMZ	72	3
11	30/M	Left temporal	AA	III	IDH mutant	2018: Gross Total excision	2019: EBRT 45 Gy 25 # booster 14.4 Gy 8 # with concurrent TMZ	24	6
12	39/M	Left inferior frontal	AA	III	IDH wildtype	2018: Gross Total excision	2018-2019 EBRT 60 Gy 30 # with concurrent TMZ	1	12
13	34/F	Right frontal	AODG	III	IDH mutant	2018: Decompression	2019: EBRT 59.4Gy in 33 # with concurrent TMZ	12	5
14	16/M	Left thalamus	Midline glioma	IV	H3K27 mutant	2019: Partial excision and decompression	2019: TMZ followed by EBRT: 49.5 Gy 33 #	1	9
15	29/M	Right parietal	GBM	IV	IDH wildtype	2017: Decompression 2018: Not operable	2017: EBRT 60Gy in 30 # with concurrent TMZ 2018: TMZ	18	4
16	42/F	Left frontal	AA	III	IDH mutant	2016: Gross Total excision	2016: EBRT 60 Gy 30 # with concurrent TMZ	32	3
17	51/F	Left temporal	GBM	IV	IDH wildtype	2018: Gross Total excision	2018: 2016: EBRT 60 Gy 30 # with concurrent TMZ	2	10
18	29/F	Right parietal	GBM	IV	NOS(R132H negative)	2018: Gross Total excision	2019: IMRT 59.5 Gy 33 # with concurrent TMZ	1	7
19	40/M	Left temporal	GBM	IV	NOS(R132H negative)	2018: Near total excision 2019: Gross Total excision	2018: EBRT 54.4 Gy 33 # with concurrent TMZ	13	1
20	8/F	Right frontoparietal	High grade glioma	IV	IDH wildtype	2018: Gross Total resection	2018: IMRT RT 60Gy 33 # with concurrent TMZ	20	3
21	16/M	Right Frontal	Spindle cell Sarcoma	III	NA	2016: Near total excision	2018: EBRT 54.4 Gy 33 # with concurrent TMZ		
22	29/F	Left frontal	AA	III	IDH wildtype	2017: Gross Total excision	2017: EBRT 60Gy 33 # with concurrent TMZ	24	10
23	44/M	Right frontal	AA	III	IDH mutant	2016: Gross total excision	2016: EBRT 60Gy 30 # with concurrent TMZ	33	9
24	45/F	Right temporoparietal	AODG; Final-Anaplastic ependymoma	III	IDH wildtype, L1CAM positive	2017: Gross total excision	2017: EBRT 60Gy 30 # with concurrent TMZ	24	1
25	32/F	Left temporoparietal	AODG	III	IDH mutant	2018: Near Total excision	2018 EBRT 60Gy 30 # with concurrent TMZ	12	3
26	35/F	Left parietal	AODG	III	R132H negative	2017: Decompression with near total excision	2018: EBRT 60Gy 30 # with concurrent and adjuvant TMZ	8	7
27	31/M	Left frontoinsular	AODG	III	IDH mutant	2019: Subtotal excision n	2019: EBRT 60Gy 30 # with concurrent and adjuvant TMZ (on going)	1	3
28	44/M	Right frontal and corpus callosum	ODG	II	NA	2014: Subtotal excision	2014: Incomplete treatment EBRT 10 # 2019: 6 cycles of TMZ	60	8
29	48/M	Right parietooccipital	AA	III	IDH mutant	2017: Near Total excision	2017: EBRT 60Gy 30 # with concurrent TMZ	29	7
30	28/M	Left temporal	GBM	IV	IDH wildtype	2015: Subtotal excision	2016: EBRT 60Gy 30 # with concurrent TMZ	59	1
31	59/M	Right temporal	GBM	IV	IDH wildtype	2019: Gross total excision 2020: Recurrence- Gross total resection	2019: EBRT 60Gy 30 # with concurrent TMZ 2020: Adjuvant TMZ	4	1
32	47/M	Left temporoparietal	AODG	III	IDH mutant	2015: Gross total excision	2016: EBRT 60Gy 30 # with concurrent TMZ	48	6
33	30/M	Corpus callosum	GBM	IV	IDH wildtype	2016: Partial resection	2016:EBRT 43 Gy in 23 # with concurrent TMZ	40	1
34	31/M	Bifrontal	AODG	III	IDH mutant	2018: Near total excision	2019: EBRT 60 Gy in 30 #, with concurrent TMZ	11	5
35	35/M	Right parietal	ODG	II	IDH mutant	2016: Gross Total excision	2016: EBRT 55.8 Gy in 30# no chemotherapy	40	4
36	40/M	Right frontal	AA	III	IDH mutant	2019: Gross Total excision	2019: EBRT 60 Gy in 30 #, with concurrent TMZ	7	7
37	43/F	Right parietal	AODG	III	NA	2013: Subtotal excision	2013; EBRT 60 Gy in 30 #, NO chemotherapy received, lost to follow-up for 6 years	78	5
38	68/M	Left frontal	GBM	IV	IDH wildtype	2019: Gross total excision	2020: EBRT 60 Gy in 30 #, with concurrent TMZ	1	4
39	32/F	Right frontal	AODG	III	IDHR132H neg	2018: Gross Total excision	2018: EBRT 60 Gy in 33 cycles#, with concurrent TMZ	15	4
40	51/F	Right frontal	AODG	III	IDH mutant	2018: Gross Total excision	2018: EBRT 60 Gy in 33 cycles#, with concurrent TMZ	22	4
41	36/M	Right frontal	AA	III	IDH mutant	2018: Gross Total excision	2018: EBRT 60 Gy in 30 cycles#, with concurrent TMZ	17	13
42	57/M	Right frontal	AODG	III	IDH mutant	2018: Gross Total excision	2018: EBRT 60 Gy in 30 cycles#, with concurrent TMZ	10	13
43	36/F	Left frontoparietal	GBM	IV	IDH mutant	2009: Gross Total excision 2016: Recurrence- re-exploration subtotal excision	2009: EBRT60 Gy in 30 cycles#, with concurrent TMZ 2016: EBRT alone with no chemotherapy 2017: Bevacizumab	24	13
44	34/M	Right temporoparietal	AODG	III	IDH mutant	2018: Near total excision 2019: Re exploration subtotal excision	2018: 60 Gy in 30 cycles#, with concurrent TMZ	2	14
45	69/F	Right temporal	GBM	IV	IDH wildtype	2019: Gross total excision	2019: 60 Gy in 30 cycles#, with concurrent TMZ	6	1
46	51/M	Left temporal	AODG	III	IDH wildtype	2019: Gross total excision	2019: 60 Gy in 30 cycles#, with concurrent TMZ	3	13
47	50/M	Left parietal	GBM	IV	IDH wildtype	2018: Gross total excision	2018: EBRT RT 60 Gy in 30 cycles#, lost to follow-up	12	12
48	43/F	Right parietal	AODG	III	NA	2017: Gross total excision	2018: EBRT RT 60 Gy in 30 cycles#, lost to follow-up	78	7

*GBM* glioblastoma multiforme, *AA* anaplastic astrocytoma, *AODG* anaplastic oligodendro glioma, *ODG* oligodendroglioma. Size of the tumour could not be measured because all 11C PET MRI were performed post chemoRT; hence, anatomical distortion on MRI images to give relevant sizes (MRI Volumetric analysis not done)

The sensitivity, specificity, positive predictive value, negative predictive value, and diagnostic accuracy for each parameter using an appropriate threshold value determined from the coordinates of the ROC curve are shown in Table 3.

**Fig. 4:**
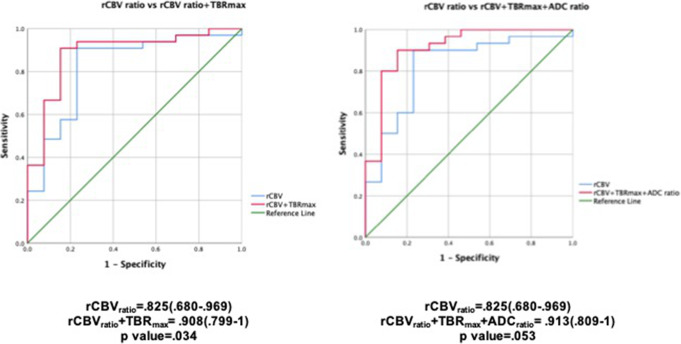
ROC curves for relative CBV and TBR_max_ combined compared to relative CBV alone is shown on the left. The graph on the right shows the ROC curve for relative CBV with TBR_max_ and ADC_ratio_ compared to relative CBV alone. The area under curve for rCBV and TBR_max_ taken together increased to 0.908 compared to 0.825 for rCBV alone with statistically significant difference. The area further increased to 0.913 with ADC_ratio_ without reaching statistical significance

A combined ROC curve analysis was performed with rCBV ratio in combination with TBR_max_ and rCBV along with TBR_max_ and ADC ratio. The AUC for rCBV + TBR_max_ was higher than that of rCBV alone (0.908 vs. 0.823) showing higher diagnostic accuracy which was statistically significant (*p* = 0.034). Adding ADC ratio further increased the AUC to 0.913; however, the difference was not statistically significant (Fig. 4).

### Agreement of PET, MR perfusion, and diffusion diagnosis on visual assessment with the final diagnosis

Using the Cohen kappa statistic, substantial agreement was seen between PET and the final diagnosis (kappa = 0.766) with moderate agreement of rCBV maps (0.472), ADC maps (0.499), and post-contrast images (0.451).

### Concordance between PET, MR perfusion, and diffusion

**Fig. 5:**
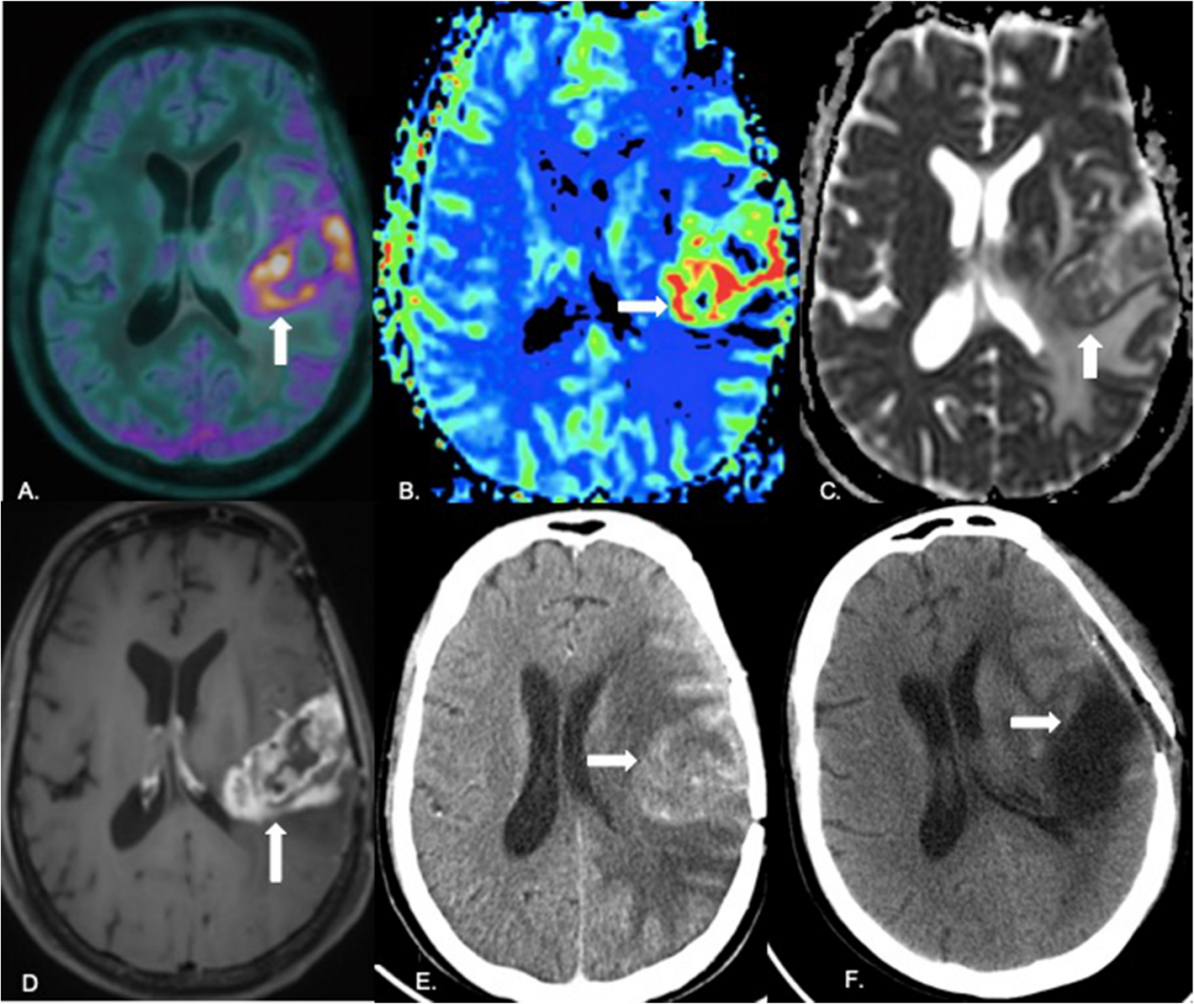
Concordant PET, perfusion, diffusion and enhancement in a case of recurrence: 40-year-old male diagnosed with anaplastic ODG 15 months back. Underwent surgical excision followed by radiotherapy completed 13 months earlier. PET image overlaid over T1 MPRAGE (**A**) shows a large area of uptake in the left temporoparietal region (white arrow). rCBV map (**B**) generated with the leakage correction algorithm shows elevated perfusion in the same region (white arrow) with diffusion restriction on the ADC map (white arrow in **C**). Note enhancement on the post-contrast image (white arrow in **D**). In view of unequivocal evidence of recurrence, surgery was planned. Post-contrast axial CT brain acquired prior to surgery (**E**) shows an enhancing left parietal lesion (white arrow). Post-operative plain axial CT brain (F) shows the resection cavity (white arrow) with gross total excision. Histopathology revealed recurrent GBM. This is a case of recurrence where the PET, perfusion, diffusion, and contrast images were concordant with good spatial congruence

**Fig. 6:**
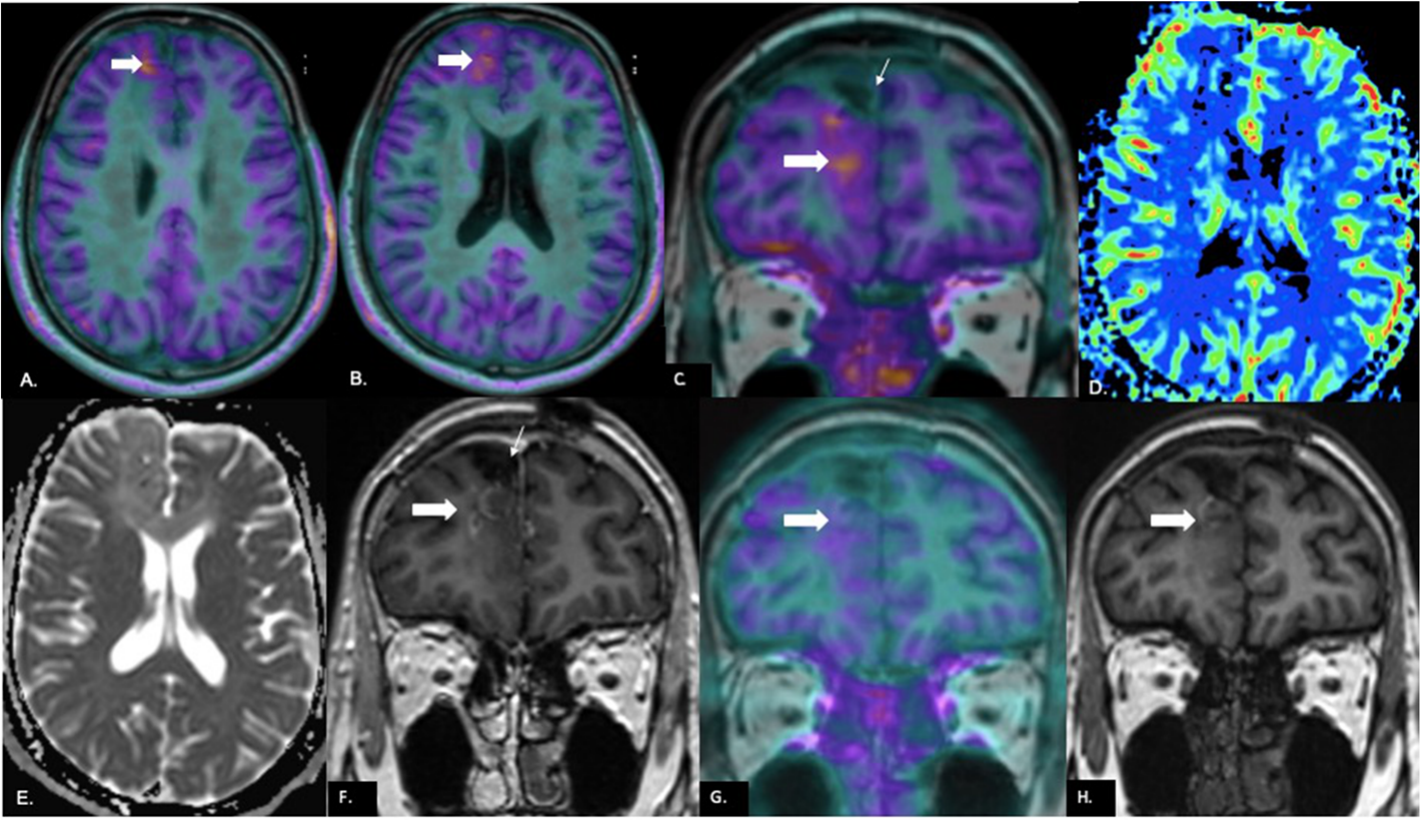
Discordance between PET, perfusion and diffusion in a case of recurrence: 31-year-old male operated for right frontal ODG 14 months back; last radiation dose 10 months ago. PET images fused with axial T1 MPRAGE (**A**, **B**) show an area of uptake in the right superior frontal gyrus (white arrow in **A**, **B**). The uptake (block white arrow) is seen along the margins of the resection cavity (small white arrow) on the coronal fused PET-MR image (**C**). rCBV map (**D**) generated using the leakage correction algorithm shows no unequivocal elevation of perfusion in the area. There is no diffusion restriction on the ADC map (**E**). Few foci of enhancement (block white arrow) are seen along the margins of the resection cavity (small white arrow) on post-contrast coronal T1 MPRAGE (**F**). In view of PET uptake, recurrence was suspected and chemotherapy administered. Follow-up PET image after chemotherapy fused with coronal T1 MPRAGE (**G**) shows reduction in the degree of uptake (block white arrow) along the resection cavity margin when compared with prechemo coronal PET image in **C**. Reduction in the degree of enhancement is also seen on the follow-up post-contrast coronal T1 MPRAGE (white arrow in **H**) compared to that in **F**

**Fig. 7:**
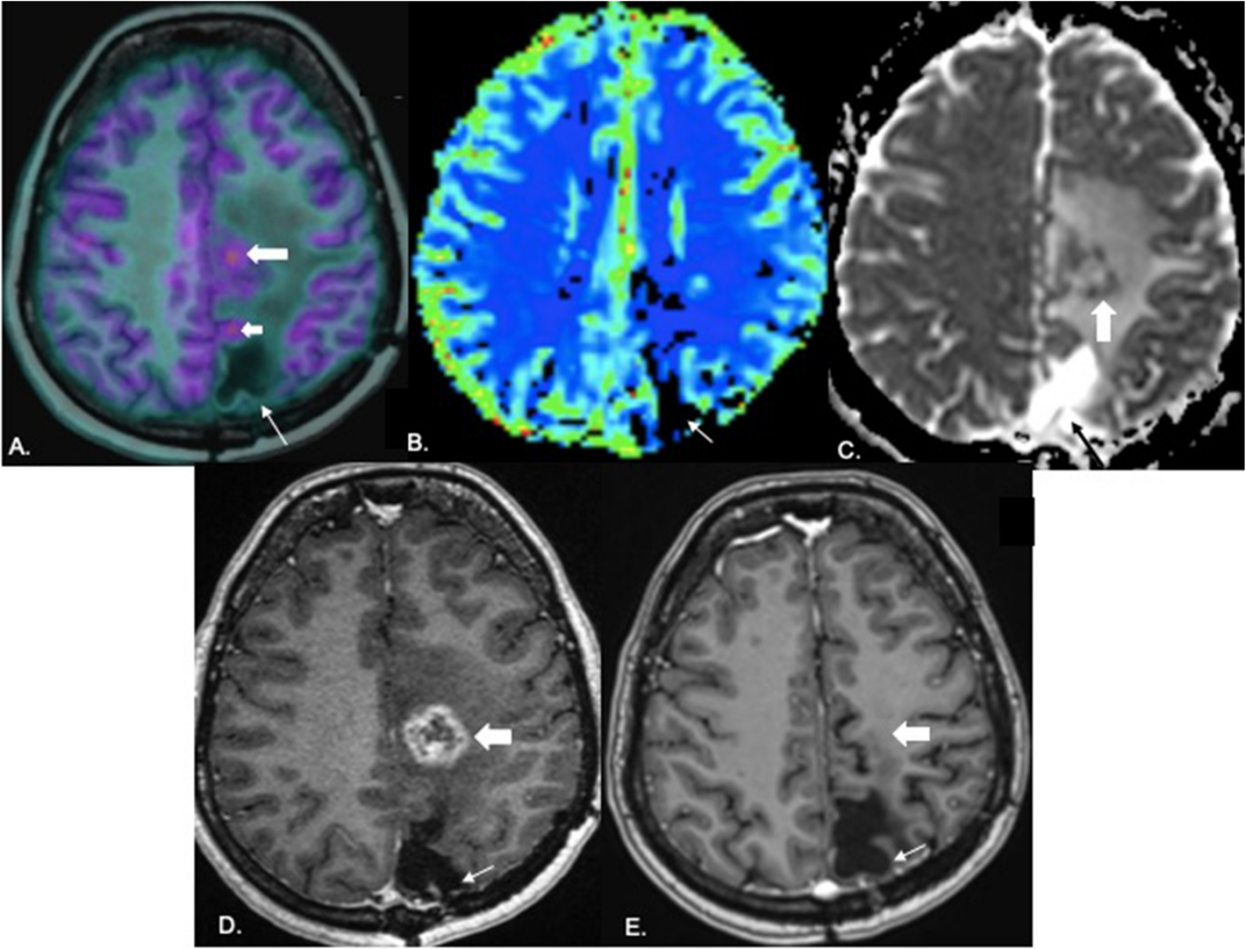
False positive diagnosis on PET: 34-year-old female diagnosed with anaplastic ODG 2 years back with enhancing lesion on MRI. PET image fused with axial T1 MPRAGE (**A**) shows an area of uptake along the parasagittal region of the left frontal lobe (large thick arrow) and another area (small thick arrow) along the margin of the resection cavity (linear white arrow) raising a suspicion of recurrence. There is no evidence of elevated perfusion on the rCBV map (**B**). Resection cavity is shown by white arrow. Peripheral diffusion restriction is seen along the margins of the lesion in the left centrum semiovale (block white arrow in **C**). There is no restriction along the resection cavity (black arrow). Peripheral enhancement is seen along the margins of the lesion in the left centrum semiovale (block white arrow in **D**) with no enhancement along the resection cavity (white arrow). One month follow-up axial post-contrast T1 MPRAGE (**E**) shows the resection cavity (thin white arrow) with complete resolution of enhancement in the left centrum semiovale (block white arrow) suggestive of treatment related changes excluding recurrence

There was discordance between ADC and PET in 11 cases (22.9%), between rCBV and PET in 8 cases (16.7%), and between PET and contrast enhancement in 7 (14.6%) cases (Fig. 5). In the case of 11 cases with diffusion-PET discordance, PET correctly classified 9 out of 11 cases as recurrence (Fig. 6). Out of 8 cases of discordance between perfusion and PET, only one was incorrectly classified as recurrence on PET while in 7 cases of PET-contrast enhancement discordance, one case was misclassified on PET as recurrence. All instances of incorrect classification on PET were a result of false positive diagnosis in which radiation necrosis was diagnosed as recurrence (Fig. 7). Area of PET uptake and elevated perfusion on MRI showed partial (less than 50%) and near complete (more than 50%) concordance in 12.5 and 70.9% cases respectively. On ADC maps, partial and near complete concordance of area of diffusion restriction with PET uptake was seen in 14.6 and 62.5% cases respectively. There was 16.7% partial and 68.8% near complete concordance between contrast enhancement and PET uptake.

## Discussion

Differentiating recurrence from post-treatment changes in gliomas is critical for patient management. The former requires re-exploration and resection of the tumor, while the latter may be managed medically with steroids. Besides radiation necrosis, other treatment-related changes like pseudo-progression and pseudo-response add to the diagnostic confusion. Radiation necrosis occurs around 3 to 12 months following radiotherapy and presents as an increase in post-contrast enhancement on conventional MRI (Verma et al., 2013). Pseudoprogression results from exaggerated response to treatment and is generally seen within 3 months following radiotherapy with or without chemotherapy. Pseudoresponse occurs due to reduced vascularity following treatment with the anti-angiogenic drug bevacizumab resulting in a decrease in enhancement in an otherwise viable tumor (Zikou et al., 2018).

Conventional magnetic resonance imaging, commonly used for post-treatment follow up of gliomas, falters in differentiating recurrence from treatment related changes since both recurrences, early and delayed radiation changes show an increase in enhancement on post-contrast images secondary to disruption of the blood brain barrier (Verma et al., 2013; van Dijken et al., 2019). MR perfusion, diffusion, and molecular imaging techniques provide surrogate markers for angiogenesis and cell proliferation which are features of recurrent tumor (Ronca et al., 2017). Several studies have been performed using these techniques in isolation (Wang et al., 2015; Nihashi et al., 2013; van Dijken et al., 2019; Barajas Jr et al., 2009) and combination (Seeger et al., 2013; Keunen et al., 2014; Heiss et al., 2011; Jena et al., 2017; Nael et al., 2018) with variable results.

**Fig. 8:**
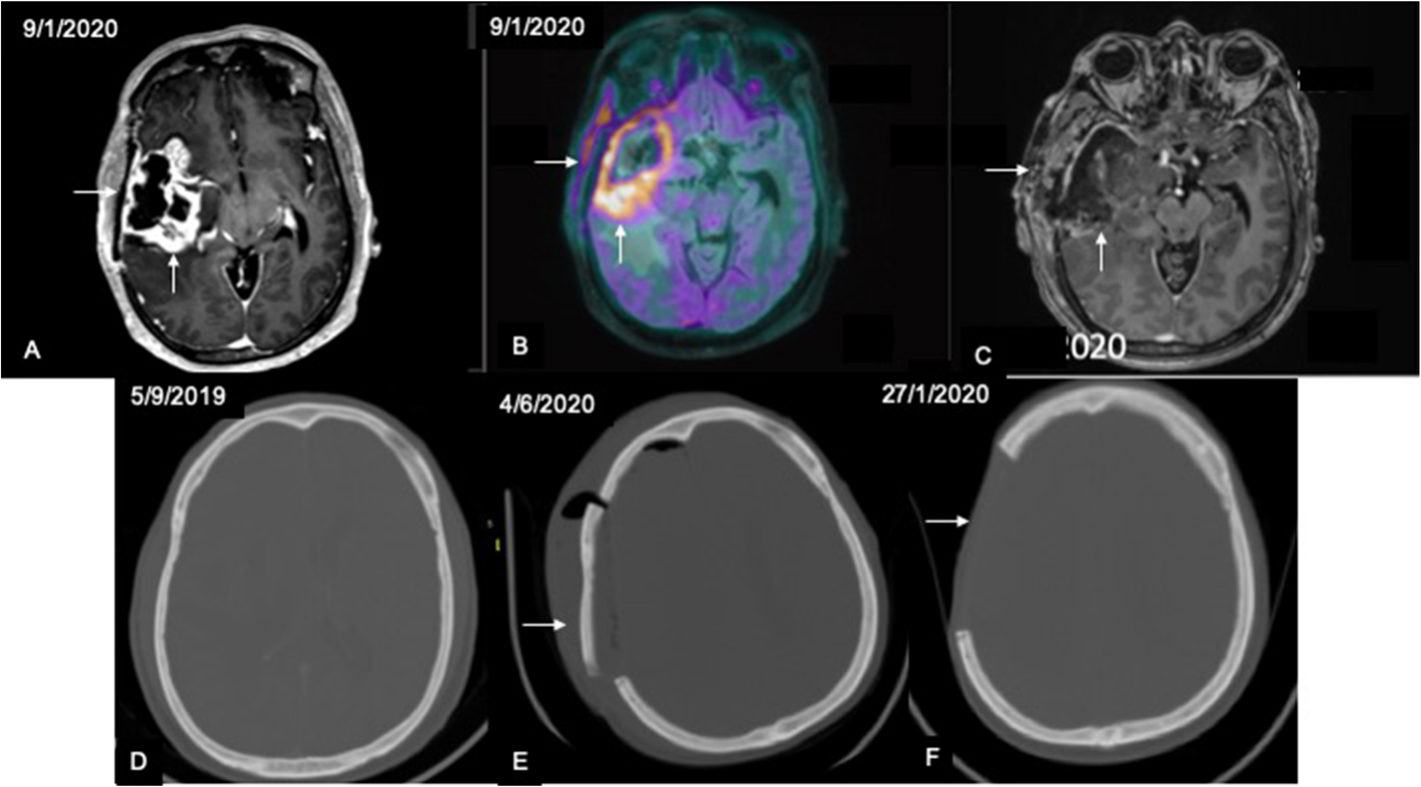
Sequence of images showing false positive C11 methionine uptake in acute inflammatory pathology: Axial post-contrast T1 MPRAGE (**A**) in a case of suspected recurrence shows parenchymal enhancement (vertical arrow) suggestive of recurrent lesion with associated enhancement of the overlying bone and soft tissues (horizontal arrow). C11 methionine PET image fused with axial T1 MPRAGE (**B**) shows tracer uptake along the bone and soft tissues (horizontal arrow) in addition to parenchymal uptake (vertical arrow). Here the uptake in the bone and soft tissues occurred due to osteomyelitis. Follow-up axial post-contrast T1 MPRAGE following chemo/radiotherapy (**C**) shows reduction in parenchymal enhancement (vertical arrow) with persistent enhancement in bone and soft tissues (horizontal arrow). Sequential axial CT brain images in bone window (**D**–**F**) show initial scan (**D**) with normal bone followed by progressive osteomyelitis in **E** and excision of the infected bone in **F**

Molecular imaging is a useful adjunct to advanced MRI in identifying recurrence in post-treatment gliomas. Most of the PET studies have been performed using 18-fluoro-deoxyglucose (FDG) as the radiotracer. Although some studies reported a low specificity for FDG-PET in detecting recurrence (Ricci et al., 1998; Hustinx et al., 2005), a meta-analysis by Wang et al. (Wang et al., 2015) revealed diagnostic performance comparable to magnetic resonance spectroscopy (MRS). In this metanalysis comparing FDG-PET, C-11 methionine PET, and MRS, the pooled sensitivity for FDG-PET was the lowest at 70% and the specificity was highest at 88% (Wang et al., 2015). The heterogeneity in data related to FDG-PET and lower sensitivity may partly be attributed to the uptake of FDG by the normal brain parenchyma which leads to misdiagnosis in some cases (Soni et al., 2020). In this regard, amino acid tracers such as C-11 Methionine have a distinct advantage in that the background normal parenchymal uptake is much less thus leading to a higher tumor to background ratio and has shown promising results in various studies (Takenaka et al., 2014; Kim et al., 2010; Deuschl et al., 2018; Minamimoto et al., 2015; Terakawa et al., 2008). In our study, the highest AUC and highest diagnostic accuracy were obtained for TBR_max_ with a sensitivity slightly less than that of rCBV ratio (81.8 vs. 84.8%) and a much higher specificity (92.3 vs. 76.9%) using a threshold of 1.23. These results are comparable to those of other studies which have reported sensitivity and specificity ranging between 66–91 and 60–100% respectively for differentiating recurrent lesion from treatment related changes (Takenaka et al., 2014; Kim et al., 2010; Deuschl et al., 2018; Minamimoto et al., 2015; Terakawa et al., 2008). A metanalysis (Nihashi et al., 2013) revealed pooled sensitivity of 70% and specificity of 93% for detection of recurrence in high grade gliomas using C11-methionine PET which is comparable to the values obtained in this study. Besides, we also observed that visual analysis of PET images for recurrence showed the highest agreement with the final diagnosis compared to that of MR perfusion and diffusion. This is expected in view of the increased contrast between lesion uptake and brain parenchyma even in lesions located close to the cortex. Our findings are corroborated by another study (Minamimoto et al., 2015) in which no significant difference was seen between visual and quantitative analysis in differentiating recurrent brain lesions from radiation necrosis on C11-methionine PET. However, one of the important disadvantages of C11-methionine is that uptake may also be seen in areas of radiation necrosis (Fig. 7) and acute inflammatory pathology (Fig. 8) leading to a false positive diagnosis (Ogawa et al., 1991). In view of uptake by inflammatory cells, it has been reported to show lower diagnostic accuracy compared to other amino acid radiotracers like fluroethyltyrosine (FET) and fluoro-dihydroxyphenylalanine (F-DOPA) (Nihashi et al., 2013; Minamimoto et al., 2015; Galldiks et al., n.d.). In this cohort, we encountered a false positive diagnosis in 4 out of 48 cases (8.33%) on methionine PET in which the findings were discordant with MR perfusion and diffusion.

MR perfusion has an established role in the detection of recurrent disease. Perfusion parameters are markers of neovascularization which characterizes progressive disease. DSC-perfusion is the most common method used and has been studied in greater detail compared to other methods like dynamic contrast-enhanced (DCE) perfusion and arterial spin labeling (ASL) (van Dijken et al., 2019). Quantitative assessment of perfusion maps especially rCBV and rCBV ratio calculated in relation to the contralateral normal parenchyma have shown good diagnostic yield in several studies (Barajas Jr et al., 2009; Nael et al., 2018; Wang et al., 2016). We obtained a sensitivity and specificity of 84.8 and 76.9% using a cut-off value of 1.38 for rCBV ratio to detect recurrence. Our results are comparable to a pooled analysis which showed sensitivity ranging between 82 and 91% and specificity between 77 and 91% for the detection of recurrence using DSC perfusion (van Dijken et al., 2019). However, the threshold rCBV ratio shows wide variation among studies ranging between 0.71 and 3.7 (van Dijken et al., 2019). This may be due to co-existence of recurrent disease and radiation necrosis (Blasel et al., 2016) and leakage across the blood brain barrier leading to errors in estimation since DSC perfusion works on the premise that the contrast agent is confined to the intravascular compartment (van Dijken et al., 2019). We tried to overcome this limitation to some extent by using a leakage correction algorithm to process the perfusion study. Visual assessment of rCBV maps showed only moderate agreement with the final diagnosis which was much less than that seen with amino acid PET. This may be accounted for by the fact that small areas of elevated perfusion or lesions near the cortex are likely to be overlooked in view of the background normally perfused brain parenchyma unlike PET using amino acid tracers where an area of uptake distinctly stands out from the rest of the normal brain. Thus, while diagnostic accuracy of quantitative PET and perfusion parameters are comparable, visual assessment of amino acid PET does score over rCBV maps in detecting recurrence, however, at a cost of false positive diagnosis in some cases.

High cell density and continued cellular proliferation in recurrent tumor restricts the diffusion of water molecules seen as a reduction in the ADC values. However, diffusion imaging is limited by the heterogeneity of lesions leading to a relatively poor diagnostic performance (Soni et al., 2020; Brandes et al., 2008). Among the two quantitative diffusion parameters evaluated in this study, mean ADC and ADC ratio, higher AUC was obtained for ADC ratio with a sensitivity and specificity of 78.1 and 69.2% respectively for detecting recurrent tumor with threshold of 1.11. Using the visual grading scale, grades higher than grade 1 were sensitive (84%) for detecting recurrent disease at the cost of very low specificity of 46.2%. The diagnostic accuracy was 73.65% comparable with other studies (Jena et al., 2017; Nael et al., 2018). Also, the threshold ADC ratio of 1.11 shows that the ADC in recurrent lesions is nearly the same as that of the normal white matter thus making visual detection of recurrence on diffusion images difficult. Co-existence of areas of necrosis, recurrent tumor, edema and hemorrhage reflects as marked heterogeneity on diffusion images making it difficult to arrive at a specific diagnosis.

Given the different aspects of tumor biology reflected by each of these imaging modalities, it is intuitive that a combination of parameters provides a more comprehensive picture of the lesional and perilesional milieu resulting in better diagnostic performance. This is supported by the findings of our study which showed a significantly higher AUC for a combination of TBR_max_ and rCBV ratio (0.908) compared to rCBV ratio alone (0.823) thus improving diagnostic accuracy. The AUC further increased with the addition of ADC ratio (0.913); however, the difference was not statistically significant. Various studies have shown the superiority of combined PET and MRI over either modality alone (Jena et al., 2017; Kim et al., 2010; Ozsunar et al., 2010; Qiao et al., 2019). Adding TBR_max_ to choline/creatine ratio (Cho/Cr), rCBV ratio and mean ADC led to an increase in the AUC to 0.932 from 0.913 for MRI alone in a study by Jena et al (Jena et al., 2017). The best diagnostic performance was seen with a combination of TBR_mean_, mean ADC, and Cho/Cr (AUC = .935). Our results are concordant with a recent study on C11-Methionine PET with DSC perfusion where they obtained an AUC of 0.953 by combining TBR_max_ with rCBV (Qiao et al., 2019). With the advent of hybrid PET-MRI scanners, combined use of structural and functional MRI with metabolic imaging is emerging as an attractive paradigm for evaluation of post-treatment gliomas. It allows simultaneous or sequential acquisition in a single sitting thus reducing time, overcoming logistic hurdles and improving patient convenience and cooperation. Simultaneous acquisition in the same time frame allows better correlation between the dynamics of various functional parameters like uptake on PET and perfusion on MRI. With combined imaging, there are less chances of a false negative diagnosis. As was seen in our study, in cases with discordance between PET and perfusion and/or diffusion on visual analysis, there was no instance of a false negative diagnosis as all cases with recurrence showed uptake on PET. However, false positive diagnosis of recurrence was made in few cases on PET due to uptake seen in radiation necrosis as well.

Elevated perfusion and lower ADC on MRI showed moderate spatial concordance with PET uptake in 70.9 and 62.5% cases respectively. A one is to one spatial correlation is almost never seen. Discordance rate between DSC perfusion and PET for detection of recurrence in our study was 22.9% which is comparable to the rates reported in earlier studies (Hatzoglou et al., 2016; Seligman et al., 2019). A hybrid PET-MRI study on brain tumors with FET as radiotracer showed poor spatial congruence between PET uptake and elevated perfusion (Filss et al., 2014). Lack of spatial congruence brings to light the fact that areas of increased metabolic uptake on PET and elevated perfusion on MRI represent different aspects of tumor physiology and that elevated perfusion does not directly translate to hypermetabolism and vice versa. The two may co-exist in some regions with variability in rest of the lesion.

Our study had various limitations. It was a retrospective study from a single center. The sample sizes in the groups of recurrence and post-treatment changes without recurrence were discrepant. We did not have histopathology for all the cases as biopsy in cases of treatment related changes raises ethical issues. In addition, surgical decompression may not be considered in all cases of recurrence in view of poor Karnofsky performance score in many patients. Also, tumor segmentation may provide more robust results compared to co-registration alone. In addition, in view of the heterogenous nature of these lesions, ROI analysis alone from a given area without an integrative method like histogram analysis is not representative of the entire lesion.

## Conclusion

Methionine-PET and DSC perfusion are comparable for detecting recurrence in post-treatment gliomas. Diffusion MRI shows lower diagnostic accuracy in view of lesional heterogeneity. Combined PET-MR imaging with C11-methionine as tracer shows superiority over either modality alone and is a feasible option for post-treatment follow-up of gliomas. PET scores over perfusion as well as diffusion MRI in visually detecting recurrence without quantification. One is to one spatial congruence between the modalities is rarely seen as they reflect different aspects of tumor biology.

## Data Availability

The data used in the study can be provided if required on request from the corresponding author.
